# Circulating microRNAs and therapy-associated cardiac events in HER2-positive breast cancer patients: an exploratory analysis from NeoALTTO

**DOI:** 10.1007/s10549-024-07299-6

**Published:** 2024-04-30

**Authors:** S. Pizzamiglio, C. M. Ciniselli, E. de Azambuja, D. Agbor-tarh, A. Moreno-Aspitia, T. M. Suter, A. Trama, M. C. De Santis, L. De Cecco, M. V. Iorio, M. Silvestri, G. Pruneri, P. Verderio, S. Di Cosimo

**Affiliations:** 1 Unit of Bioinformatics and Biostatistics, Fondazione IRCCS Istituto Nazionale dei Tumori di Milano, Milan, Italy; 2 Department of Medical Oncology, Institut Jules Bordet and L’Université Libre de Bruxelles (U.L.B), Brussels, Belgium; 3 Frontier Science, Scotland Ltd, Kingussie, UK; 4 Mayo Clinic, Jacksonville, FL USA; 5 Swiss Cardiovascular Center, University Hospital Bern, Inselspital, Bern, Switzerland; 6 Unit of Evaluative Epidemiology, Fondazione IRCCS Istituto Nazionale dei Tumori di Milano, Milan, Italy; 7 Department of Radiation Oncology, Fondazione IRCCS Istituto Nazionale dei Tumori di Milano, Milan, Italy; 8 Unit of Molecular Mechanisms, Department of Research, Fondazione IRCCS Istituto Nazionale dei Tumori di Milano, Milan, Italy; 9 Unit of Microenvironment and Biomarkers of Solid Tumors, Department of Experimental Oncology, Fondazione IRCCS Istituto Nazionale dei Tumori di Milano, Milan, Italy; 10 Department of Advanced Diagnostics, Fondazione IRCCS Istituto Nazionale dei Tumori, Milan, Italy

**Keywords:** Circulating microRNA, HER2, Breast cancer, Cardiotoxicity, miRNAs

## Abstract

**Purpose:**

The relevance of cardiotoxicity in the context of HER2-positive breast cancer is likely to increase with increasing patient treatment exposure, number of treatment lines, and prolonged survival. Circulating biomarkers to early identify patients at risk of cardiotoxicity could allow personalized treatment and follow-up measures. The aim of this study is to examine the relationship between circulating microRNAs and adverse cardiac events in HER2-positive breast cancer patients.

**Methods:**

We based our work on plasma samples from NeoALTTO trial obtained at baseline, after 2 weeks of anti-HER2 therapy, and immediately before surgery. Eleven patients experienced either a symptomatic or asymptomatic cardiac event. Circulating microRNAs were profiled in all patients presenting a cardiac event (case) and in an equal number of matched patients free of reported cardiac events (controls) using microRNA-Ready-to-Use PCR (Human panel I + II). Sensitivity analyses were performed by increasing the number of controls to 1:2 and 1:3. Normalized microRNA expression levels were compared between cases and controls using the non-parametric Kruskal–Wallis test.

**Results:**

Eight circulating microRNAs resulted differentially expressed after 2 weeks of anti-HER2 therapy between patients experiencing or not a cardiac event. Specifically, the expression of miR-125b-5p, miR-409-3p, miR-15a-5p, miR-423-5p, miR-148a-3p, miR-99a-5p, and miR-320b increased in plasma of cases as compared to controls, while the expression of miR-642a-5p decreases. Functional enrichment analysis revealed that all these microRNAs were involved in cardiomyocyte adrenergic signaling pathway.

**Conclusion:**

This study provides proof of concept that circulating microRNAs tested soon after treatment start could serve as biomarkers of cardiotoxicity in a very early stage in breast cancer patients receiving anti-HER2 therapy.

**Supplementary Information:**

The online version contains supplementary material available at 10.1007/s10549-024-07299-6.

## Introduction

Human epidermal growth factor receptor 2 (HER2)-targeted therapies are used in clinical practice to treat both early and advanced HER2-positive breast cancer patients [1, 2]. Cardiotoxicity remains the main adverse event associated with anti-HER2 therapy especially with concomitant or sequential anthracycline-based chemotherapy [3]. Currently, cardiotoxicity is clinically assessed by regular evaluation of left ventricular ejection fraction (LVEF) by imaging techniques, mostly echocardiography [4]. However, cardiac events are generally diagnosed when a functional impairment (LVEF drop) has already occurred, not allowing for preventive strategies. The development of biomarkers to early identify patients at risk of cardiotoxicity is of the utmost importance to tailor healthcare to each individual by an optimal use of imaging, therapeutics, and follow-up.

MicroRNAs (miRNAs) are a class of non-coding 17–25 nucleotide-long single-stranded RNAs that modulate gene expression posttranscriptionally by base-pairing targeting of messenger RNAs. miRNAs play essential roles in basic biological functions, including cell proliferation and differentiation, invasion, angiogenesis, and apoptosis, and contribute to the development and fine-tuning of innate and adaptive immune responses [5]. Therefore, circulating miRNAs may represent promising biomarkers for the early and minimally invasive diagnosis of cancer and its treatment-related cellular toxicity [6–11]. Expressed in cardiac and inflammatory cells, miRNAs regulate several biological pathways triggering the left ventricular remodeling and leading to cardiac dysfunction and heart failure, hence their biomarker potential role [12, 13]. miRNAs are also released into circulation, where they are stable owing to encapsulation in extracellular vesicles or by binding to transport proteins [14]. Therefore, circulating miRNAs are protected from degradation and can be reliably measured in blood samples [15]. Recently, experimental studies have shown that circulating miRNAs can be invoked as cell–cell communication regulators and paracrine signaling mediators in left ventricular modeling [16]. The aim of the present study was to analyze circulating miRNAs with respect to cardiotoxicity in HER2-positive breast cancer patients treated with either trastuzumab, lapatinib, or their combination.

## Methods

### Study design and participants

We performed a case–control study on patients from the NeoALTTO trial (ClinicalTrials.gov registration no. NCT00553358) [17]. This trial included a prospective cohort of 455 patients with HER2-positive breast cancer who were randomized to preoperative lapatinib (*n* = 154), trastuzumab (*n* = 149), or their combination (*n* = 152) given for 6 weeks followed by same anti-HER2 therapy in combination with weekly paclitaxel for 12 weeks. After surgery, patients received three cycles of fluorouracil, epirubicin, and cyclophosphamide every 3 weeks, followed by the assigned preoperative anti-HER2 treatment for additional 34 weeks. The NeoALTTO protocol excluded patients with known history of uncontrolled or symptomatic angina, clinically significant arrhythmias, congestive heart failure, transmural myocardial infarction, uncontrolled hypertension (≥ 180/110), unstable diabetes mellitus, dyspnea at rest, or chronic therapy with oxygen (https://clinicaltrials.gov/study/NCT00553358). Adequate baseline LVEF ≥ 50% was required at study entry. Cardiac function was closely monitored with either echocardiography or multiple gate acquisition scan (MUGA) throughout the trial across all three arms at the following pre-specified timepoints: baseline, week 6 of neoadjuvant phase, pre-surgery, weeks 1, 13, 25, and 34 of adjuvant phase, months 12, 18, 24, 36, 48, and 60 of follow-up [18]. As per NeoALTTO protocol, study patients were identified as having a primary cardiac event if they experienced cardiac death and/or symptomatic congestive heart failure (CHF) according to the New York Heart Association (NYHA) class III or IV or a secondary cardiac event in case of asymptomatic (NYHA class I) or mildly symptomatic (NYHA class II) significant drop in LVEF (i.e., to below 50% and > 10 points) confirmed by a second LVEF assessment within approximately 3 weeks.

In the current study, once identified patients experiencing a cardiac event and with ≥ 1 available plasma sample, one control was randomly selected from NeoALTTO patients who remained free of reported cardiac events during the follow-up period. These control participants were matched to case participants by age, treatment arm, and primary tumor estrogen receptor (ER) status. Patient participation in the NeoALTTO study was allowed after signing the main study consent form, which included a non-specific clause for use of blood samples for biomarker research. The Internal Review and Ethics Boards of Fondazione IRCCS Istituto Nazionale dei Tumori (Milan, Italy) approved the INT 186–13 protocol for the current circulating miRNA analyses.

## Circulating miRNA profiling data pre-processing

For the aim of the present study circulating miRNA profile was analyzed on plasma samples obtained at baseline (T0), after 2 weeks of anti-HER2 treatment alone (T1), and at week 18 immediately before surgery (T2). Procedures for plasma preparation and RNA isolation have been already reported [11]. Briefly, a total of 752 miRNAs were profiled using microRNA Ready-to-Use PCR and Human panel I + II in each sample. The relative quantity (RQ) of each miRNA was calculated using the comparative threshold cycle method [19] following the formula RQ = 2^^‐Δ*Cq*^, where Δ*Cq* = (*Cq* miRNA − *Cq* reference); *Cq* reference was computed by considering the *Cq* average of all the detected miRNAs [20].

## Data analysis

For the statistical analysis, the RQ of each miRNA was considered in logarithmic scale (log2 RQ). Differentially expressed miRNAs between patients experiencing or not a cardiac event were identified by resorting to non-parametric Kruskal–Wallis test at each specific time point, i.e., T0, T1, and T2. For each of miRNA the fold change was computed by considering the median expression value in plasma of patients experiencing cardiac event (cases) *versus* the median expression value in plasma of patients not an experiencing cardiac event (controls). Statistical analysis was carried out with SAS software (Version 9.4.; SAS Institute, Inc., Cary, NC) by adopting a significance *α* level of 5%. Given the exploratory hypothesis-generating nature of our study multiplicity adjustment was not applied. Sensitivity analyses were performed by increasing the number of controls to 1:2 and 1:3. Prediction of target site of circulating miRNA(s) of interest was performed using miRWalk 3.0 [21]. Functional enrichment of circulating miRNA-targeted genes for Gene Ontology (GO) biological process terms and KEGG pathways was performed using the ClusterProfiler Bioconductor R package [22] and a false discovery rate (FDR) < 0.05 was applied to identify statistically significant enriched pathway. Network representation was performed using Cytoscape 3.9.1 [23].

## Results

### Study population and miRNAs profile

During the NeoALTTO trial (median follow-up of 6.7 years and interquartile range 5.7–6.8), a total of 13 cardiac events occurred (4 symptomatic and 9 asymptomatic) in 11 patients (2 patients treated with trastuzumab, 2 with lapatinib and 7 with their combination). Cardiac events occurred at a minimum of 4 months to a maximum of 33 months from randomization. Specifically, the cumulative incidence was 2.1% (standard error [SE] of 0.007) at 1 year and 2.6% (SE 0.008) at 3 years from randomization.

Nine of these patients had ≥ 1 plasma sample collected (either at baseline, 2 weeks after treatment or before surgery) and thus were eligible for evaluation. Specifically, plasma samples were available at all three time points for 3 patients, at two time points for 4 patients, and at one time point for 2 patients (Supplementary Table S1). Patient and primary tumor characteristics of cases developing a cardiac event and of their 9 matched controls are reported in Table 1. No changes were observed among the analyzed groups in relation to the values of the classical markers of cardiomyocyte damage and heart failure TnT and proBNP (Supplementary Table S2).

**Table 1:** Baseline characteristics of study patient population

Baseline characteristics	Controls* (*N* = 9)		Cases** (*N* = 9)	
Age	*N*	%	*N*	%
< 50	4	44.44	4	44.44
≥ 50	5	55.56	5	55.56
Body mass index				
Median (range)	28	(20–36)	25	(19–32)
Cardiovascular disorders^a^				
No	5	55.56	5	55.56
Yes	4	44.44	4	44.44
Hypertension	2	22.22	2	22.22
Arrhythmia	0	0	1	11.11
Deep vein thrombosis	0	0	1	11.11
Heart valve incompetence	1	11.11	0	0
Palpitations	1	11.11	0	0
Metabolic disorders^b^				
No	7	77.78	6	66.67
Yes	2	22.22	3	33.33
Diabetes mellitus	0	0	3	33.33
Hypercholesterolemia	2	22.22	0	0
Tumor size				
≤ 5	4	44.44	4	44.44
> 5	5	55.56	5	55.56
Nodal status				
No	2	22.22	2	22.22
Other	7	77.78	7	77.78
Estrogen receptor				
Negative	3	33.33	3	33.33
Positive	6	66.67	6	66.67
Treatment				
Lapatinib or trastuzumab	3	33.33	3	33.33
Lapatinib and trastuzumab	6	66.67	6	66.67

^a^Hypertension (2 controls and 2 cases), arrhythmia (1 case), deep vein thrombosis (1 case), heart valve incompetence (1 control), and palpitations (1 controls)

^b^Diabetes mellitus (3 cases) and hypercholesterolemia (2 controls)

^*^Controls: patients not experiencing a cardiac event; **Case: patients experiencing a cardiac event

## Association of circulating miRNAs levels with cardiac events

**Fig. 1:**
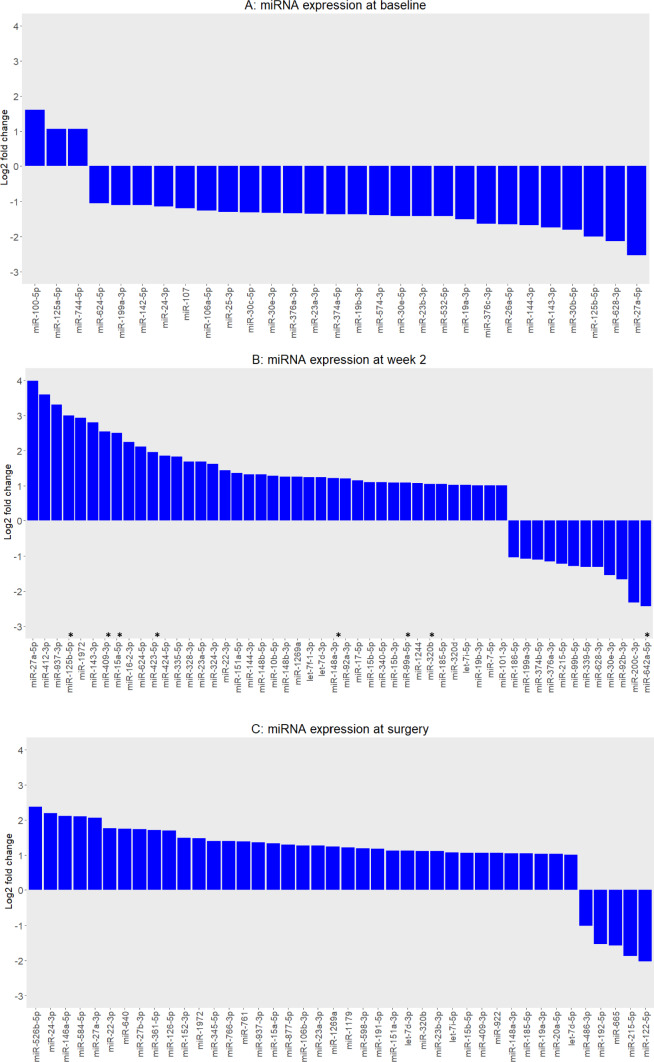
Fold changes in circulating miRNAs in patients developing a cardiac event compared to matched controls**.** Each panel reports fold change values (log2 scale) between patients experiencing or not a cardiac event of miRNAs with an absolute log2 fold change > 1 (fold change > 2 for up-regulated and < 0.5 for down-regulated miRNAs): at baseline (*N* = 29 miRNAs), after 2 weeks of anti-HER2 treatment alone (*N* = 52 miRNAs) and at week 18 immediately before surgery (*N* = 42 miRNAs). No miRNAs overlapped across the three time points. *Differentially expressed circulating miRNAs in patients experiencing or not a cardiac event

**Fig. 2:**
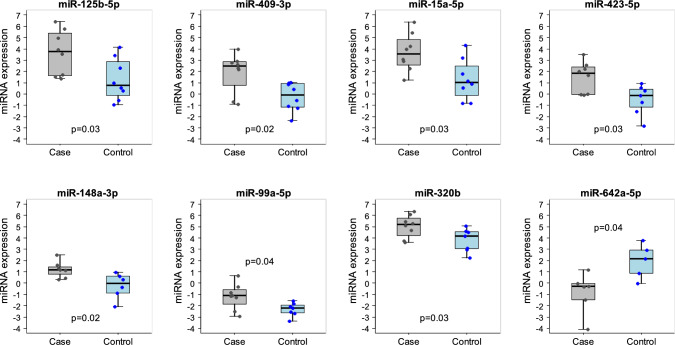
Distribution of the expression levels of cardiotoxicity-associated miRNAs. Box plot of the eight circulating miRNAs with a significant differential expression between patients experiencing (case) or not (control) a cardiac event evaluated in the plasma sample collected after two weeks of anti-HER2 neoadjuvant treatment alone. Each box indicates the 25th and 75th percentiles. The horizontal lines inside the box indicate the median, and whiskers indicate the extreme measured values; individual values are represented by dots. *p* = *p*-value of the Kruskal–Wallis test. miRNA expression = log2 (RQ)

The association between miRNA expression values and the occurrence of a cardiac event was assessed at each specific time point. In particular, a total of 138,172, and 104 miRNAs were detected in at least 5 patients with and without a cardiac event at baseline, two weeks after treatment, or before surgery, respectively. By considering the fold change (FC), we obtained a set of 29, 52, and 42 miRNAs with a FC > 2 (for up-regulated miRNAs) or < 0.5 (for down-regulated miRNAs) at baseline, at two weeks after treatment, and before surgery, respectively (Fig. 1 and Supplementary Table S3). Out of these miRNAs, none was significantly dysregulated at baseline or at the end of neoadjuvant therapy. However, a set of eight circulating miRNAs were differentially expressed after two weeks of neoadjuvant therapy treatment between patients experiencing or not a cardiac event (Fig. 2)**.** Specifically, miR-125b-5p, miR-409-3p, miR-15a-5p, miR-423-5p, miR-148a-3p, miR-99a-5p, and miR-320b were significantly up-regulated in patients with a cardiac event (fold change from 2 to 8); while miR-642a-5p was down-regulated with a fold change of 0.19. Supplementary Table S4 report descriptive statistics for the expression value of each of the 8 miRNAs according to cardiac event status. To address the limitation of the small sample size, we conducted a sensitivity analysis by employing a 1:2 and 1:3 matching control approach. It is worth noting that all initially identified miRNAs were confirmed, except for miR-642a-5p, which exhibited the lowest detection rate (< 70%) among all the analyzed miRNAs. Panels A and B of Supplementary Figure S1 illustrate the distribution of expression levels of cardiotoxicity-associated miRNAs in the 1:2 and 1:3 matching, respectively**.**

## Functional-related pathways of cardiotoxicity-related miRNAs

**Fig. 3:**
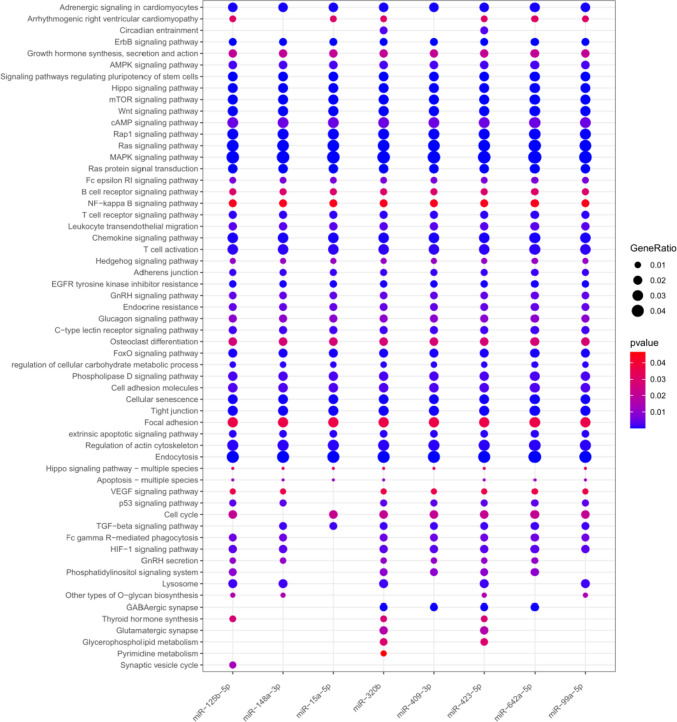
Functional enrichment analysis of targeted genes of cardiotoxicity-associated miRNAs. The size of each dot represents the miRNA-targeted gene number ratio for the corresponding pathway, whereas the shade of color from red to blue indicates *p*-values of increasing significance. The graph shows 58 statistically significant GO (*p* < 0.01) and KEGG pathways (*p* < 0.05)

miRWalk analysis returned a total of 10,754 unique genes as predicted targets of the 8 miRNAs. Specifically, 2193 genes for miR-125b-5p, 857 genes for miR-148a, 972 for miR-15a-5p, 4856 for miR-320b, 1616 genes for miR-409-3p, 6104 genes for miR-423-5p, 2474 genes for miR-642a-5p, and 681 genes for miR-99a-5p. The results of the functional enrichment analysis showed that miRNAs associated with cardiotoxicity were mostly involved in common signaling, revealing the presence of multiple functional modules. Indeed, our analysis identified 38 out 58 (65.5%) pathways characterized by a high number of shared genes (range = 39–277; median = 99.5) directly influenced by miRNAs (FDR *p*-value < 0.05), including PI3K/Akt, ERBB, and Ras signaling, immune response, and metabolic processes (Fig. 3 and Supplementary Figure S2). On the contrary, the non-common pathways were characterized by a low rate of affected genes (range = 21–88; median = 57) with only synaptic vesicle cycle and pyrimidine metabolism signaling specifically associated to single miRNAs (miR-125b-5p and miR-320b, respectively) (Supplementary Table S5). The functional enrichment analysis, finally, highlighted how all miRNAs were unanimously impinging on the pathway of adrenergic signaling in cardiomyocytes, directly affecting 102 out of 4404 genes composing the pathway (Fig. 3 and Supplementary Table S5).

## Discussion

In this work, we performed a time-course profile of circulating miRNAs in plasma samples collected during the neoadjuvant treatment of patients who experienced a cardiac event in the NeoALTTO trial and of an equal number of matched controls. For the best of our knowledge, this is the first report showing an association between miRNA expression profile already after two weeks of treatment and later cardiotoxicity in patients treated with anti-HER2 therapy. A total of eight miRNAs, namely miR-125b-5p, miR-409-3p, miR-15a-5p, miR-423-5p, miR-148a-3p, miR-99a-5p, miR-320b, and miR-642a-5p, in the plasma samples collected soon after starting treatment identified patients who later experienced adverse cardiac events at a median follow-up of about 7 years. Of note, miR-642a-5p has to be considered with caution due to its low detection rate (< 70%). The relationship between these miRNAs and specific biological pathways suggests their involvement in adrenergic signaling in cardiomyocytes. Intriguingly, none of the miRNAs tested at baseline and after treatment were significantly associated with cardiac events, suggesting that circulating miRNAs could be regarded as time-sensitive cardiac biomarkers.

Reversible suppression of HER2 signaling in cardiomyocytes is the main mechanism leading to cardiotoxicity in patients receiving anti-HER2 therapy [24]. More recently, preclinical studies showing that trastuzumab may cause ultrastructural alternations in heart tissues have questioned the reversibility concept of trastuzumab-associated cardiotoxicity. In addition, subclinical damage associated with anti-HER2 could become clinically manifest in the long-term [25]. In the absence of level 1 data on the type and frequency of cardiac monitoring and its impact on patient outcomes, oncologists and cardiologists should adhere as closely as possible to current guidelines [26, 27]. This is to protect patients undergoing new and effective anti-HER2 therapies that increase both survival and exposure to potentially toxic drugs. Specifically, overall cardiotoxicity profile of dual HER2 blockade is safe [28]. However, a recently reported pooled analysis showed that dual blockade with pertuzumab was associated with an increased risk of heart failure compared with trastuzumab (Relative Risk = 4.18, 95% Confidence Interval: 1.07–16.30) [29]. As these data should not be overlooked in the context of early-stage disease, development of biomarkers to predict for cardiotoxicity and allowing prompt identification and treatment of patients at risk of cardiac events is of the utmost importance. Elevated serum biomarkers have been associated with anthracycline-related toxicity; however, the predictive and prognostic value of troponin T, troponin I, and BNP monitoring remains controversial [30, 31]. Of note, in the NeoALTTO trial neither troponin T nor BNP were useful to predict cardiotoxicity [18].

The present study showed that eight miRNAs were differentially expressed according to the occurrence or not of a cardiac event. Some of these miRNAs were already described in other settings (Supplementary Table S6). miR-423-5p is up-regulated by anthracyclines [32] and is both diagnostic and prognostic in patients with CHF [33, 34]. miR-125b is significantly higher in the blood of patients with acute stroke compared to controls [35, 36] and is exquisitely detected in cardioembolic cases [37]. The miR-15 family, including miR-15a, miR-15b, miR-16, miR-195, miR-497, and miR-322, is abundantly expressed in cardiac cells [38], where its up-regulation acts as an endogenous inhibitor of extracellular matrix remodeling by targeting TGF-βR1 and the downstream targets p38, SMAD3, SMAD7, and endoglin [39]. miR-148a expression changes dynamically in distinct subtypes of heart failure, resulting elevated in concentric hypertrophy and decreased in dilated cardiomyopathy [40]. miR-148a alleviates cardiac dysfunction, immune disorders, and myocardial apoptosis in myocardial ischemia–reperfusion injury by targeting pyruvate dehydrogenase kinase 4 [41]. miR-99a negatively regulates physiological hypertrophy through mTOR signaling [42]. Finally, miR-409-3p is significantly down-regulated in the sera of patients with mitral regurgitation developing heart failure compared to controls [43]. Consistent with these data, our enrichment analysis showed private pathways for each cardiac miRNA. Even more intriguingly, all these miRNAs seem to affect the adrenergic signaling. A long time now, several studies reported alterations in the cardiac β-receptor system in failing hearts (reviewed in 44). Specifically, the β1 subtype and its mRNA down-regulations correlate with severity, while β2 receptor levels remain unchanged in most studies. The significance of β1 changes and stability of β2 in CHF is debated. They may be interpreted as beneficial mechanisms that protect from the damaging effects of β-receptor stimulation, including arrhythmias, energy imbalance, hypertrophy, and apoptosis. Alternatively, they may lead to further deterioration of heart failure as they prevent the heart from meeting its demands [44]. Clinically, β-adrenergic antagonists represented one of the most important advances in the treatment of patients with CHF, but it is still debated whether they act by blocking or by re-sensitizing the β-adrenergic receptor system.

Our study includes several differences and certain advantages over previous research. First, we observed the occurrence of adverse cardiac events over a relatively long period of follow-up. Second, all blood samples and LVEF evaluations were performed according to the NeoALTTO schedule as early as 2 and 6 weeks after treatment, conferring an increased chance of recording pathophysiologic changes in the early stages of cardiotoxicity. Intriguingly, at a variance of week 2 profile, miRNAs tested at baseline and after neoadjuvant therapy were uninformative. We can speculate that miRNAs at baseline are influenced by an untreated patient and does not necessarily imply a direct relationship with later cardiotoxicity caused by the treatment intended to use. miRNAs assessed early during treatment reflect a different situation represented by the encounter of delivered HER2-targeted agent, the host, and their balance, thus indicating a possible predictive value. In addition, most of miRNAs found are certainly expressed by cardiac cells, but its circulating levels might be the result of the contribution of other different cell types, including immune components, known to be relevant for both HER2-targeted therapy efficacy and toxicity [45]. The lack of informative value of miRNAs tested after treatment may be due to the interference of chemotherapy that might alter circulating miRNA levels. Thus, rather than evaluating absolute levels of single miRNAs in patients with unknown individual backgrounds, comorbidities, and lifestyle, our findings suggest that to evaluate the expression patterns of miRNAs early during the course of treatment to catch the drug/host interplay.

We acknowledge that our study is limited by its retrospective nature and the characteristics inherent in case–control research. It is important to note the relatively small number of patients with a cardiac event in our cohort. However, this is expected not greatly affecting our results because there were no significant differences in baseline characteristics between cases and controls thanks to the adopted matching design. At the same time, to compensate for this limitation, we will continue to verify the prognostic value of the set of miRNAs generated in NeoALTTO in an independent external cohort that we are establishing, although not enough patients have been followed until now. Another issue is represented by the qRT-PCR methodology used in this study that does not allow for easy and rapid application in a clinical setting. However, further development of technology might overcome this limitation in future. Moreover, the normalization strategies for miRNA expression data should involve a combination of endogenous and exogenous normalization methods, an approach that should be considered in the next studies.

## Conclusion

Using a time-course high-throughput profiling, this study shows that increased levels of circulating miR-125b-5p, 409-3p, 15a-5p, 423-5p, 148a-3p, 99a-5p, and 320b and decreased levels of miR-642a-5p are associated with adverse cardiac events. These miRNAs might aid in prediction of cardiotoxicity at a very early stage in patients receiving anti-HER2 therapy.

### Supplementary Information

Below is the link to the electronic supplementary material.

Supplementary file1 (7Z 104 KB)

## Data Availability

The raw data supporting the conclusions of this article will be made available by the authors upon reasonable request.
